# HLA Class II Allele, Haplotype, and Genotype Associations with Type 1 Diabetes in Benin: A Pilot Study

**DOI:** 10.1155/2017/6053764

**Published:** 2017-07-20

**Authors:** Kaossarath A. Fagbemi, Thierry C. Marc Medehouenou, Simon Azonbakin, Marius Adjagba, Razack Osseni, Jocelyne Ahoueya, Arnaud Agbanlinsou, Raphael Darboux, Lamine Baba-Moussa, Anatole Laleye

**Affiliations:** ^1^UFR de Biologie Humaine, Faculté des Sciences de la Santé, Université d'Abomey-Calavi, Cotonou, Benin; ^2^Laboratoire de Recherche en Biologie Appliquée, Ecole Polytechnique d'Abomey-Calavi, Université d'Abomey-Calavi, Abomey-Calavi, Benin; ^3^Laboratoire de Biologie et de Typage Moléculaire en Microbiologie, Université d'Abomey-Calavi, Abomey-Calavi, Benin

## Abstract

**Background:**

Several studies have reported the implication of HLA-DR/DQ loci in the susceptibility to type 1 diabetes (T1D). Since no such study has yet been performed in Benin, this pilot one aimed at assessing HLA class II allele, haplotype, and genotype associations with T1D.

**Material and Methods:**

Class II HLA genotyping was performed in 51 patients with T1D and 51 healthy unrelated controls by means of the PCR-SSP method. The diagnosis of T1D was set up according to American Diabetes Association criteria. Odds ratio (OR) and its 95% confidence interval (95% CI) were calculated to assess the associations between T1D and HLA alleles, haplotypes, and genotypes.

**Results:**

Participants were aged 1–24 years. T1D was significantly associated with DR3, DQA1∗05:01, DQB1∗02:01, and DR3-DR4. No significant associations were observed with DR4, DQB1∗03:02, and DQB1∗06:02.

**Conclusion:**

Certain HLA class II alleles, haplotypes, and genotypes were related to T1D and may be used as genetic susceptibility markers to T1D in Benin.

## 1. Introduction

Type 1 diabetes (T1D) is a chronic and autoimmune disease characterized by the destruction of insulin-producing cells [1, 2]. The incidence of T1D has been increasing worldwide by approximately 3% per year [3], with the highest increase in young children [4]. Although the burden of T1D in the youth has been emphasized in the literature during the last decade, no T1D data exist in Benin. The prevalence of all diabetes was occurring in Benin up from 1.1% in 2001 [5] to 4.6% in 2012 [6]. The implication of HLA-DR/DQ loci in the susceptibility to T1D in Caucasoid and non-Caucasoid populations has been reported [7–9]. The HLA class II region with its multiple polymorphisms is then by far the greatest contributor to the genetic predisposition to T1D [9]. HLA allele and haplotype frequencies vary considerably across ethnic groups [10] as does T1D risk [11]. However, data from many reports show that HLA-DR3, HLA-DR4, HLA-DQA1∗05:01, HLA-DQA1∗03:01, HLA-DQB1∗02:01, and HLA-DQB1∗03:02 account for HLA class II allele susceptibility to T1D whereas HLA-DR2, HLA-DQA1∗01:02, and HLA-DQB1∗06:02 are in protection to T1D [12–14].

To the best of our knowledge, few studies have investigated the implication of the HLA class II in Black African patients with T1D [15–17]. This pilot study was the first to date to assess associations of specific HLA class II alleles, haplotypes, and genotypes with T1D in Benin.

## 2. Material and Methods

### 2.1. Design and Study Sample

The present study was designed as a 1-1-matched case-control study. The participants were 51 patients with T1D attending the Bank of Insulin of Cotonou (a reference center for the diagnosis and the management of diabetics in Benin) from November 2012 to January 2016 and 51 age-, sex-, ethnic-, and residence area-matched unrelated healthy individuals without diabetes or a family history of diabetes recruited in the general population.

The study was approved by the Research Ethics Review Boards of University of Abomey-Calavi. All participants provided written informed consent.

### 2.2. Case Ascertainment

Patients with T1D were diagnosed by a physician on the basis of the following criteria: a fasting glycemia ≥ 1.26 g/dL, an unexplained weight loss, signs of hyperglycemia (polyuria, polydipsia, polyphagia, and asthenia), and an absolute insulin dependence. These criteria were defined according to the recommendations of the American Diabetes Association [18].

### 2.3. Participant Eligibility

All participants have to be originated from Benin. Patients with T1D should be diagnosed before the age of 30 years. Any participant was excluded in the acute phase of a serious illness requiring care or having a set of medicine interfering with the insulin sensibility (corticoids, growth hormone, and so forth).

### 2.4. Participant Characteristics

During the period from November 2012 to January 2016, a total of 102 participants were recruited (23 males and 28 females; sex ratio = 1.21). Of these, 51 were diagnosed with T1D and 51 without T1D (Table 1).

### 2.5. HLA Class II Genotyping

Each participant was invited to provide a 10 mL EDTA tube of peripheral blood, which was frozen at −20° Celsius at the Laboratory of Human Biology of the Faculty of Health Sciences of the University of Abomey-Calavi. The DNA extraction was realized according to the single-step method of RNA isolation by acid guanidinium thiocyanate-phenol-chloroform extraction [19, 20]. The DNA quality and its concentrations were determined by a Thermo Scientific Evolution 60S UV-Visible spectrophotometer.

HLA class II genotyping was performed with PCR-based sequence-specific primers (PCR-SSP) [21] in a 20 *μ*L mixture of 6.5 *μ*L of DNA (100 ng/*μ*L), 0.8 *μ*L of MgCl_2_ (2 mM)_,_ 2 *μ*L of PCR buffer 1x, 2 *μ*L of each deoxynucleotide triphosphate (200 *μ*M), 4 *μ*L of forward primers (20 *μ*M), 4 *μ*L of reverse primers (20 *μ*M), and 0.7 *μ*L of polymerase Taq 0.175 U (Invitrogen). The process of DNA amplification is described as follows: the program of amplification contains an initial denaturation of 2 min in 94°C, 32 cycles of amplification (every cycle consists of a denaturation of 30 s in 94°C, a hybridization of primers during 30 s in 63°C, and an extension of 30 s in 72°C), and a final extension of 10 min in 72°C. The products of amplification were separated on a 2% agarose gel. The sequences of primers used for the amplification of the genes are presented in Table 2.

### 2.6. Statistical Analysis

Data were collected in the MS Excel 2007. The association between T1D and each identified HLA-DR/DQ alleles, haplotypes, and genotypes was assessed using an odds ratio (OR) with its 95% confidence interval (95% CI). The SAS software (version 9.1 SAS Institute Inc., Cary, NC, USA) was used to perform all statistical analyses.

## 3. Results and Discussion

### 3.1. Results

The association between T1D and specific HLA class II alleles, haplotypes, and genotypes was assessed using an odds ratio (Table 3). Significant associations were found between T1D and DR3 haplotype (OR = 13.3; 95% CI: 4.37–40.6), DQA1∗05:01 (OR = 03; 95% CI: 1.37–6.55) and DQB1∗02:01 (OR = 2.4; 95% CI: 1.06–5.31) alleles, and DR3-DR4 genotype (OR = 12; 95% CI: 2.00–71.9). In contrast, no significant associations were found between T1D and DR4 haplotype (OR = 02; 95% CI: 0.64–6.24) and DQB1∗03:02 (OR = 0.80; 95% CI: 0.27–2.07) and DQB1∗06:02 (OR = 0.65; 95% CI: 0.22–1.82) alleles.

### 3.2. Discussion

The present study is a pilot one in Benin focusing on the association between T1D and HLA genotyping with the sequence-specific primers of DR3, DR4, DQA1∗05:01, DQB1∗02:01, DQB1∗03:02, and DQB1∗06:02. These preliminary data showed a light predominance of female sex. A similar result was found by Timoteo et al. [22]. In contrast, EURODIAB data showed 1.06 as the sex ratio in favor of male sex [23]. The analyses of HLA disease associations in different ethnic populations, due to differences in allele frequency distributions and patterns of linkage disequilibrium, can allow important general inferences of disease risk associated with specific alleles and with specific combinations of alleles. Then, T1D has been strongly associated with HLA class II alleles in a variety of populations. Beside the marginal protective effects of DQB1∗03:02 and DQB1∗06:02 alleles observed in this study, DR3 haplotype, DQA1∗05:01, and DQB1∗02:01 alleles, and DR3-DR4 genotype showed positive significant associations with T1D. These latter alleles, haplotypes, and genotypes might be seen as T1D risk markers. Our results also indicated that DR3 haplotype, DQA1∗05:01 and DQB1∗02:01 alleles, and DR3-DR4 genotype increased T1D risk by >2 to ≤14 times in Beninese.

These results, together, showed that there is a relationship between the presence of these HLA class II alleles, haplotypes, and genotypes and the risk of developing T1D in Beninese as in Caucasoid population [8]. Indeed, Fajardy et al. showed positive associations between DQA1∗05:01 allele and the T1D onset among French children [24]. Similar results with DR3 haplotype and DQA1∗05:01 allele were also reported in the Lithuanian [25], in the Brazilian [26], and in the Spanish [27] populations.

The T1D risk was not significantly associated with DR4 haplotype and DQB1∗03:02 and DQB1∗06:02 alleles in the present study. In contrast, it was reported in the literature that there were positive associations between T1D and DR4 haplotype [8] and DQB1∗03:02 allele [8, 28]. A protective effect of DQB1∗06:02 allele in T1D was also observed [29].

## 4. Conclusion

This study is one of the first to assess the associations of HLA class II alleles, haplotypes, and genotypes with the risk of developing T1D in Benin. Certain HLA class II alleles, haplotypes, and genotypes were related to T1D and may be used as genetic susceptibility markers to T1D. Further studies of HLA and T1D in Benin are needed to confirm the present results and to provide data for the development of screening assays and for better management of patients with T1D at the onset disease.

## Figures and Tables

**Table 1:** Ages of study participants.

Age range	Patients with T1D	Controls
	*n* (%)	*n* (%)
<5	04 (7.8)	04 (7.8)
5–15	39 (76.5)	39 (76.5)
>15	08 (15.7)	08 (15.7)

**Table 2:** Sequences of the couples of primers used for the amplification of the genes.

Primers	Sequences
HLA-DR3	Fw: 5′CACGTTTCTTGGAGTAC3′ Rv: 5′CGTAGTTGTGTCTGCAGTAGT3′
HLA-DR4	Fw: 5′CAGGTTAAACATGAGTGTCATTTCTTAAAC3′ Rv: 5′GCTGTCGAAGCGCACGTACTCCTCTTGGTG3′
HLA-DQA1∗05:01	Fw: 5′ACGGTCCCTCTGGCCAGTA3′ Rv: 5′AGTTGGAGCGTTTAATCAGAC3′
HLA-DQB1∗02:01	Fw: 5′GTGCGTCTTGTGAGCAGAAG3′ Rv: 5′GCAAGGTCGTGCGGAGCT3′
HLA-DQB1∗06:02	Fw: 5′CGTGCGTCTTGTGACCAGAT3′ Rv: 5′GCTGTTCCAGTACTCGGCAT3′
HLA-DQB1∗03:02/3	Fw: 5′GACGGAGCGCGTGCGTTA3′ Rv: 5′AGTACTCGGCGTCAGGCG3′
HLA-DQB1∗03:03	Fw: 5′GACGGAGCGCGTGCGTTA3′ Rv: 5′CTGTTCCAGTACTCGGCGT3′

Fw: forward; Rv: reverse.

**Table 3:** Associations between T1D and HLA class II alleles, haplotypes, and genotypes.

HLA alleles, haplotypes, and genotypes	Participants		ORs (CI 95%)
	Cases (*n* = 51)	Controls (*n* = 51)	
DR3	42	05	13.3 (4.37–40.6)
DR4	15	11	02 (0.64–6.24)
DQB1∗02:01	26	15	2.4 (1.06–5.31)
DQB1∗03:02	16	18	0.75 (0.27–2.07)
DQB1∗06:02	10	13	0.65 (0.22–1.82)
DQA1∗05:01	35	19	03 (1.37–6.55)
DR3-DR4	13	02	12 (2.00–71.9)

OR: odds ratio; 95% CI: 95% confidence interval.
